# Comment on: “Indirect Assessment of Skeletal Muscle Glycogen Content in Professional Soccer Players Before and After a Match Through a Non-Invasive Ultrasound Technology *Nutrients *2020, *12*(4), 971”

**DOI:** 10.3390/nu12072070

**Published:** 2020-07-13

**Authors:** Niels Ørtenblad, Joachim Nielsen, Kasper D. Gejl, Harry E. Routledge, James P. Morton, Graeme L. Close, David C. Niemann, Julia L. Bone, Louise M. Burke

**Affiliations:** 1 Department of Sports Science and Clinical Biomechanics, University of Southern Denmark, 5230 Odense, Denmark; jnielsen@health.sdu.dk (J.N.); kgejl@health.sdu.dk (K.D.G.); 2 Research Institute for Sport and Exercise Sciences, Liverpool John Moores University, Liverpool L3 3AF, UK; routledge.nutrition@gmail.com (H.E.R.); J.P.Morton@ljmu.ac.uk (J.P.M.); G.L.Close@ljmu.ac.uk (G.L.C.); 3 Department of Biology, Appalachian State University, Bonne, NC 28608, USA; niemandc@appstate.edu; 4 Australian Catholic University, Melbourne, Fitzroy Vic 3065, Australia; julia.bone@gmail.com (J.L.B.); louise.burke@acu.edu.au (L.M.B.)

San-Millán and colleagues [1] present data on muscle glycogen content captured by ultrasound technology. Although a simple, noninvasive technique is attractive, we question the validity of this approach. MuscleSound^®^ converts pixilation intensities in ultrasound images to a score of glycogen content based on muscle water content. In our view, there are no experimental or theoretical bases for such a link. We acknowledge that pixilation intensity may be related to muscle water content, and that glycogen particles have an osmotic effect on intracellular water. However, the athlete’s hydration status affects muscle water content independently of glycogen [2]. Furthermore, glycogen-depleting high-intensity exercise is accompanied by an osmotically driven increase in muscle water due to accumulation of intracellular lactate ions and metabolites [3]. The manipulation of muscle creatine content may also affect water content [4]. In all cases, as is relatively common in sport, glycogen and water content change independently or in opposite directions.

Only two studies have been conducted by independent research groups (i.e., without financial interests in MuscleSound^®^), and both found no associations between muscle glycogen content determined by biopsies and MuscleSound^®^ score [5,6]. San-Millan and colleagues argue that the relatively small muscle samples analysed from biopsies cannot correlate with the MuscleSound^®^ score of glycogen based on the entire muscle. This appears to be circular reasoning, as they simultaneously argue that the MuscleSound^®^ score is evaluated by correlating with histological and biochemical estimations of glycogen from the same site. Additionally, the argument contrasts with experimental findings demonstrating little variation in glycogen content between different sites of the same muscle [7]. Furthermore, our paper [8] is used to argue that “muscle glycogen is stored in different pools within the same muscle and in different muscles according to different muscle fibres”. This represents an incorrect understanding of the subcellular glycogen localisation, which is distinct at the individual muscle fibre level (µm level), where even a small biopsy sample represents 1000s of fibres. Additionally, the notion that a 25% decrease in muscle glycogen corresponds to a ~10% decrease of SR Ca^2+^ release and uptake is a misinterpretation of another study [9], since this association only seems to occur when glycogen levels are reduced below ~50% of resting levels.

Finally, it is surprising that MuscleSound^®^ estimates report average muscle glycogen utilisation of only 20% during a competitive soccer match, with only one player experiencing >25% decrease and the rest a mean reduction of 17%. This is strikingly low compared to the 50% depletion observed from muscle biopsies [10], further questioning the methodology.

As a consequence, the MuscleSound^®^ technology has no ample experimental or theoretical bases for a possible estimation of muscle glycogen and cannot be used neither for a scientific nor applied purpose.
